# Designing an implementation strategy to improve interprofessional shared decision making in sciatica: study protocol of the DISC study

**DOI:** 10.1186/1748-5908-7-55

**Published:** 2012-06-15

**Authors:** Stefanie N Hofstede, Perla J Marang-van de Mheen, Willem JJ Assendelft, Carmen LA Vleggeert-Lankamp, Anne M Stiggelbout, Patrick CAJ Vroomen, Wilbert B van den Hout, Thea PM Vliet Vlieland, Leti van Bodegom-Vos

**Affiliations:** 1 Department of Medical Decision Making, Leiden University Medical Center, Albinusdreef 2, 2333 ZA Leiden, The Netherlands; 2 Department of Public Health and Primary Care, Leiden University Medical Center, Einthovenweg 20, 2333 ZC Leiden, The Netherlands; 3 Department of Neurosurgery, Leiden University Medical Center, Albinusdreef 2, 2333 ZA Leiden, The Netherlands; 4 Department of Neurology, University Medical Centre Groningen, Hanzeplein 1, 9700 RB Groningen, The Netherlands; 5 Department of Orthopedic Surgery, Leiden University Medical Center, Albinusdreef 2, 2333 ZA Leiden, The Netherlands

**Keywords:** Sciatica, Lumbar radicular syndrome, Implementation strategy, Shared decision making, Barriers and facilitators, Decision aid

## Abstract

**Background:**

Sciatica is a common condition worldwide that is characterized by radiating leg pain and regularly caused by a herniated disc with nerve root compression. Sciatica patients with persisting leg pain after six to eight weeks were found to have similar clinical outcomes and associated costs after prolonged conservative treatment or surgery at one year follow-up. Guidelines recommend that the team of professionals involved in sciatica care and patients jointly decide about treatment options, so-called interprofessional shared decision making (SDM). However, there are strong indications that SDM for sciatica patients is not integrated in daily practice. We designed a study aiming to explore the barriers and facilitators associated with the everyday embedding of SDM for sciatica patients. All related relevant professionals and patients are involved to develop a tailored strategy to implement SDM for sciatica patients.

**Methods:**

The study consists of two phases: identification of barriers and facilitators and development of an implementation strategy. First, barriers and facilitators are explored using semi-structured interviews among eight professionals of each (para)medical discipline involved in sciatica care (general practitioners, physical therapists, neurologists, neurosurgeons, and orthopedic surgeons). In addition, three focus groups will be conducted among patients. Second, the identified barriers and facilitators will be ranked using a questionnaire among a representative Dutch sample of 200 GPs, 200 physical therapists, 200 neurologists, all 124 neurosurgeons, 200 orthopedic surgeons, and 100 patients. A tailored team-based implementation strategy will be developed based on the results of the first phase using the principles of intervention mapping and an expert panel.

**Discussion:**

Little is known about effective strategies to increase the uptake of SDM. Most implementation strategies only target a single discipline, whereas multiple disciplines are involved in SDM among sciatica patients. The results of this study can be used as an example for implementing SDM in other patient groups receiving multidisciplinary complex care (e.g., elderly) and can be generalized to other countries with similar context, thereby contributing to a worldwide increase of SDM in preference sensitive choices.

## Background

Sciatica, more accurately called lumbosacral radicular syndrome, is a form of radiating back pain, mostly caused by a herniated disc with nerve root compression. It is characterized by radiating leg pain in combination with dermatomal motor, sensory, or tendon reflex abnormalities. Sciatica is a common condition worldwide. In Western countries 5 to 10 per 1,000 persons annually develop sciatica, with variable pain intensities and disease course [1]. In the Netherlands, sciatica patients are initially diagnosed by general practitioners (GPs) and advised to continue daily activities with or without physical therapy (conservative treatment). After a period of six to eight weeks, the leg pain diminishes in 70 % of the patients [2]. The remainder of the patients is usually referred to a neurologist for further investigation, often involving an MRI. If the MRI confirms a herniated disc, compatible with the radicular symptoms, the patient can be referred to the neurosurgeon or orthopedic surgeon to consider surgery [3,4]. In general, surgery leads to more rapid relief than prolonged conservative treatment in patients suffering radiating leg pain for more than eight weeks, but with smaller risks for prolonged conservative treatment, and both treatments have similar outcomes and societal costs at one year follow-up [5-7]. Therefore, the Dutch multidisciplinary guideline recommends that the team of professionals involved in sciatica care and patients jointly decide about treatment after this six to eight week period, i.e., surgery or prolonged conservative treatment, based on the evidence regarding associated risks and benefits and preference of the patient [8]. After all, both treatment options have equivalent results and the choice thus can be considered preference sensitive. This situation is optimally suited for interprofessional shared decision making (SDM) [9].

SDM enables patients to make an informed choice in collaboration with the professionals involved, and is important for providing care consistent with patient preferences. The Dutch government tries to make healthcare more patient-orientated, for example, by enabling free choice of insurance company, and a law that obligates professionals to discuss consequences and risks of each treatment option [10]. Despite these efforts to deliver patient-centered care, and the sciatica guideline recommendation, there are strong indications that SDM for sciatica patients is not yet widely used. Recently, a comparison between regions in the Netherlands showed considerable variation in the number of patients that undergo surgery, ranging from 31 to 140 per 100,000 inhabitants [11]. In addition, Dutch surgery rates for sciatica patients are four times higher than those in the UK and two times higher than in Sweden [11]. Only the United States have a 40 % higher surgery rate than The Netherlands [12]. This is remarkable, because the guidelines in the United States and the UK show similarities, and both suggest referring patients to a specialist when they do not respond to standard noninvasive treatment or suffer from neurological deficits [13,14]. It is very unlikely that this (inter)national variation is only caused by case mix and patient preferences. Research has shown that patients prefer a shared approach over a physician-dominated one, and are more likely to favor conservative treatments over surgery after patients’ decision aid (DA) exposure [15,16]. Furthermore, it has been shown that Dutch patients are used to delegate treatment decisions to their professionals, so that professional preferences dominate treatment decisions [17]. Thus, it is far more likely that noncompliance with the evidence-based back pain guidelines, specifically the lack of applying SDM, combined with surgeon preferences are responsible for the varying surgery rates. SDM may diminish this variation, prevent underuse and overuse of surgery [18], and thereby improve quality of care.

### Objective

The DISC study (the Dutch Implementation Study of interprofessional Shared Decision Making in Sciatica) aims to explore the barriers and facilitators associated with the everyday embedding of SDM for sciatica patients in the Dutch healthcare context, among all involved professionals and patients, and to develop a tailored, team-based, strategy for SDM implementation among sciatica patients.

## Methods

The study consists of two phases (Table 1).

A. Identification of barriers and facilitators

i. Barriers and facilitators are explored for SDM implementation

**Table 1:** Study phases and time schedule

	**Planning (months)**
**Phase A. Identification of barriers and facilitators**	
i. Barriers and facilitators are explored for SDM implementation	
Literature study and preparation interviews/focus groups	1 to 3
Interviews and focus groups	3 to 10
ii. Identified barriers and facilitators are ranked by importance in a representative sample	
Survey among professionals and patients	11 to 13
**Phase B. The development of an implementation strategy based on phase A**	
Development of the implementation strategy and expert panel	13 to 15
Writing report	16

### Study design

Barriers and facilitators among relevant stakeholders are explored in an interview study among professionals and in a focus group study among patients. The semi-structured interviews and focus groups are based on the framework developed by Grol and Wensing [19] in combination with the Normalization Process Model (NPM) [20]. The framework of Grol and Wensing [19] describes barriers and facilitators at the levels of the innovation, the professional, the patient, the social context, the organizational context, and the external environment (political and economic factors). However, the organizational context of their framework does not cover all relevant aspects for the implementation of SDM in practice. Therefore, we additionally use the NPM, which includes more details with respect to the organizational context [20] than the framework of Grol and Wensing. Normalization in the NPM is defined as the routine embedding of a complex intervention in healthcare, and this model thus offers a robust structure for investigating the collective work that leads to this embedding (or not), including:

1. Endogenous factors

a. Interactional workability: influence of SDM on interactions between people and practices.

b. Relational integration: relationship of SDM to existing knowledge and relationships.

2. Exogenous factors

a. Skill set workability: influence of SDM on current division of labor.

b. Contextual integration: relationship of SDM to the organizational setting.

The combination of the two frameworks thus ensures that all relevant aspects affecting implementation of SDM will be covered. The semi-structured interviews will be conducted among all professionals involved in the diagnosis and treatment of sciatica patients (GPs, physical therapists (PTs), neurologists, neurosurgeons, and orthopedic surgeons).

The focus group procedures of Morgan et al. will be used in preparing and conducting the focus group sessions [21]. A moderator and an observer will guide the focus groups. A group will consist of six to eight participants. When information saturation is not reached after this initial round, the focus groups will be extended in specific groups.

### Study population

We anticipate interviewing eight professionals in each of the target groups (GPs, PTs, neurologists, neurosurgeons, and orthopedic surgeons). In each group of professionals, we will continue until data saturation is reached, defined as three consecutive interviews without new ideas emerging (stopping criterion) [22]. To obtain contrasting views on barriers and facilitators, we select professionals from specific regions with either high surgery rates (most likely to raise barriers for SDM) or low surgery rates (most likely to raise facilitators for SDM) based on published reports [11,23]. In addition, we ensure diversity of gender and hospital type (public hospital and private treatment centers), because this may influence the experienced barriers and facilitators.

We anticipate organizing three focus groups, with six to eight patients in each group [24]. To create homogeneous groups, one focus group will include patients who have had surgery, one will include patients who have had conservative treatment, and one focus group will include patients that still have to decide on treatment. Patients will be recruited through advertisements in the local newspapers. When needed, additional patients will be recruited via the patient registries of GPs, neurologists, neurosurgeons, and orthopedic surgeons coordinated by the Spine Intervention Prognostic Study (SIPS) Group.

Inclusion criteria for patients are: age ≥18 years, a doctor’s diagnosis of sciatica no longer than 12 months ago, and a written informed consent. Patients with an inability to understand written and oral Dutch instructions or with active diseases likely to interfere with the purpose of this study, such as a terminal illness or severe psychiatric diseases, will be excluded from the study.

### Analysis

The semi-structured interviews and focus group interviews will be audio-taped and transcribed in full. They will be qualitatively analyzed using thematic framework analysis [25] to classify and organize data according to key themes, concepts and predefined categories. The predefined categories of the framework of Grol and Wensing will be used [19] regarding the level of the innovation, the professional, the patient, the social context, the organizational context, and the external environment (political and economic factors). We will compare the barriers and facilitators, to look for differences that may explain lack of SDM implementation. We use Atlas.ti software for analysis.

### Outcome measures

This study phase results in a list of identified barriers and facilitators for the implementation of SDM, grouped in a commonly used theoretical framework.

ii. Identified barriers and facilitators are ranked by importance in a representative sample

### Study design

**Figure 1:**
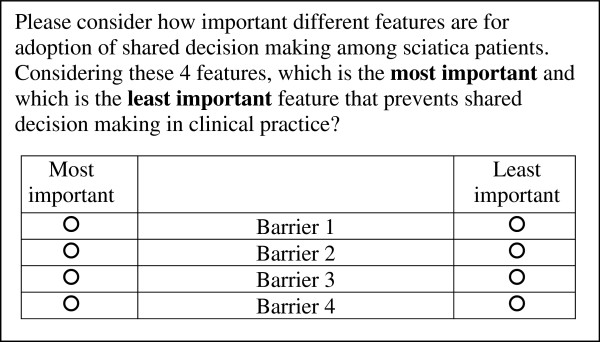
Hypothetical example of MaxDiff task.

We will conduct an internet-based questionnaire study among professionals and patients, to rank the identified barriers and facilitators from the interviews and focus groups. A maximum difference scaling (MaxDiff) exercise with an orthogonal design will be included in this questionnaire [26]. MaxDiff is a method to rank multiple items in a more efficient manner, with the additional advantage of scale-free rating so that it prevents scale use bias [27]. With this method, respondents choose the most and least important item within a set of items (Figure 1), with different sets offered to respondents a number of times.

Each set thus provides more information than a number of pairwise comparisons and forces tradeoffs between items, thereby resulting in greater discrimination. A MaxDiff task is easy to complete for participants, and results in ratio-scaled scores of importance [26,28]. The order of items will be randomized between respondents, and each item will be equally represented, to avoid higher importance given to first mentioned items.

### Study population

The survey will be sent to a representative sample of GPs (n = 200), PTs (n = 200), neurologists (n = 200), all neurosurgeons (n = 124), orthopedic surgeons (n = 200) and patients (n = 100).

The sample of professionals will be randomly selected from the Dutch medical address book and the membership lists of the professional organizations. The neurosurgeons (n = 124) are included from the same sources. We will sample patients using the patient registries of GPs, PTs, neurologists, neurosurgeons, and orthopedic surgeons, and advertisements in local newspapers.

### Analysis

Based on the choices made by respondents, importance scores will be estimated for each barrier and facilitator, for each individual respondent, using hierarchical Bayes estimation [29]. Differences between groups of respondents will be further analyzed in SPSS.

### Outcome measures

A list of the 10 most important barriers and facilitators for implementation of SDM among sciatica patients.

B: Development of a tailored implementation strategy

### Study design

The DISC study group will develop a tailored, team-based strategy to enhance the adoption of SDM. This strategy will focus on the 10 most important barriers and facilitators found in phase A. Because multifaceted strategies are more effective than single strategies [30,31] and our expectation that several barriers at different levels will be found, it is plausible that the developed implementation strategy will include several components directed at different levels. Furthermore, it is expected that the strategy components will include educational outreach, an interactive educational strategy, and/or patient-specific strategies, because these facets seem to be promising for implementation of SDM [31,32].

In the development process, the project team will use the intervention mapping approach of Bartholomew et al. [33]. This method begins with the creation of matrices, in which the performance objectives are set against the 10 most important barriers and facilitators. Subsequently, the project team will brainstorm about the strategy components needed to achieve the performance objective in the presence of the barrier or facilitator mentioned in the matrix. The cells of the matrices are then gradually filled with implementation strategy components [34]. Next, the project team will translate the formulated strategy components into practical strategies.

After the implementation strategy has been developed, an expert meeting will be held with a panel of GPs, PTs, neurologists, neurosurgeons, orthopedic surgeons, patient representatives of the Dutch back pain patients’ association, and implementation experts (n = 10 to 20) to discuss the feasibility, to refine the developed implementation strategy, and to gain acceptance of relevant stakeholders with respect to SDM.

### Analysis

The expert meeting will be audiotaped and summarized by two observers and compared until consensus is reached. The participants of the expert meeting receive a summary of the meeting and are asked whether this summary is consistent with the conclusions reached in the meeting.

### Outcome measures

A tailored strategy likely to be effective to implement SDM among sciatica patients in daily practice.

### Ethical approval

This study protocol has been presented to the Medical Ethical Committee of the Leiden University Medical Center. Ethical approval for this type of study is not required under Dutch law.

## Discussion

Implementation of SDM enables sciatica patients to make better informed decisions congruent with their preferences on whether to undergo prolonged conservative treatment or surgery. However, there are strong indications that SDM is not yet adopted in daily practice. Professional preferences seem to dominate treatment decisions, consistent with evidence that Dutch patients are used to delegate treatment decisions to their professionals [17]. Little is known about barriers and facilitators to SDM and effective strategies to increase the uptake of SDM [35]. For successful implementation of SDM in daily practice, a tailored strategy is needed focused on the barriers and facilitators of each domain influencing the adoption of SDM.

To facilitate implementation of SDM in the treatment of sciatica patients, an evidence-based guideline and a DA have already been developed. The goal of the DA was to inform sciatica patients about the two treatment options. However, this DA was not successful in stimulating SDM. This may be due to the fact that DAs are not primarily developed for use during the consultations, and thus do not necessarily stimulate SDM [18]. The extent to which the DA is used in clinical practice is unknown. Despite the Dutch multidisciplinary guideline for SDM and the availability of a DA, SDM has not been adopted in clinical practice so far. This emphasizes that barriers are likely to exist when it comes to guideline adherence and to adoption of SDM. We need to determine these barriers to develop an effective implementation strategy that is not only evidence-based, but also targets these barriers.

Known barriers to SDM reported in previous studies include time constraints and lack of applicability, due to patient characteristics or to the clinical situation [36]. However, these studies focused on implementation among one discipline only, whereas insight into barriers and facilitators for the implementation of interprofessional SDM is lacking [37], and particularly relevant for the multidisciplinary sciatica care. To our knowledge, our study will be the first to examine barriers and facilitators to interprofessional SDM. This will generate new knowledge that may also be applied among other types of patients, given that these barriers and facilitators may not be patient-specific but rather organization or context-specific.

Limitations of this study may be the selection of patients and professionals. It is possible that selection bias occurs, because professionals who are familiar with SDM in daily practice may be more motivated and willing to participate. Professionals who are not using SDM in their consultation may be less likely to participate, and may experience other barriers. To minimize the bias in the interviews, we will stratify our sampling by selecting participants from regions with respectively low and high surgery rates. Another measure taken to avoid participation bias and to yield all relevant barriers is to continue with the interviews until three consecutive interviews emerge without new ideas (stopping criterion) [22]. Similarly, selection bias may occur in the focus groups as patients with pain or other symptoms may be less likely to travel to Leiden to attend a focus group. We will minimize selection bias in the survey by sending multiple reminders to increase the response. In addition, we will test for differences between responders and non-responders in distribution of gender, hospital type, and the location of the hospital to assess whether we may generalize our findings to the total sample.

The generated knowledge and understanding of the implementation process can be used to implement SDM for sciatica patients in the Netherlands and in other countries with a similar context. Furthermore, our study can be used as an example for implementing SDM in other patient groups receiving multidisciplinary complex care such as elderly patients. Increased use of SDM may reduce referral, improve patient satisfaction [38], reduce overuse of one of the treatment options [16,18,39] and thus increase both quality and efficiency of healthcare [40,41].

## Abbreviations

DA, decision aid; GP, general practitioner; PT, physical therapist; MaxDiff, maximum difference scaling; NPM, normalization process model; SDM, shared decision making; ZonMw, The Netherlands organization for Health Research and Development.

## Competing interests

The authors declare that they have no competing interests.

## Authors’ contributions

LB and TV designed the study protocol. SH wrote the manuscript and will carry out the study. LB and PM will supervise the study and supervised writing of the manuscript. All authors have critically read and modified both the study protocol and previous drafts of the manuscript, and have approved the final version.
